# Radiographic Features of a Strangulated Transomental Hernia

**DOI:** 10.7759/cureus.62484

**Published:** 2024-06-16

**Authors:** Bao H Nguyen, Aron S Mcguirt

**Affiliations:** 1 General Surgery, University of Central Florida College of Medicine, Orlando, USA; 2 General Surgery, Bay Pines VA Health Care system, St. Petersburg, USA

**Keywords:** closed-loop obstruction, computed tomography abdomen, small-bowel resection, laparoscopic appendectomy, acquired omental defect, bowel adhesion, transomental hernia, strangulated internal hernia, small-bowel obstruction

## Abstract

This is a case report of an 82-year-old male who presented with intractable and diffuse abdominal pain and had a computed tomography (CT) abdomen showing a closed loop obstruction in the right hemiabdomen with anteromedial displacement of the cecum and ascending colon. Exploratory laparotomy revealed a gangrenous segment of the ileum strangulated by a transomental hernia in the right lower quadrant. The nonviable bowel was resected, and the healthy bowel segments were anastomosed. It is important to correlate the clinical signs of bowel obstruction with radiographic findings of internal hernia to expedite surgical intervention and prevent complications of bowel ischemia.

## Introduction

Internal hernia occurs when a segment of the bowel protrudes into an intraperitoneal structure and accounts for 3-5% of the causes of small bowel obstructions (SBOs) [1]. Among SBOs caused by internal hernias, approximately 20% require bowel resection due to gangrene [2]. Overall, internal hernias that become strangulated have a mortality rate exceeding 50% [1,2]. Transomental hernia is a subtype representing 2-3% of internal hernias, which involves protrusion of the bowel through a structural defect in the omentum [1]. Transomental hernias frequently cause SBOs and therefore carry the highest risk of bowel ischemia and gangrene [3,4]. Transomental hernias have been linked to congenital omental defects in children as well as acquired omental defects in adults who underwent major abdominal surgeries, such as bariatric surgery or liver transplantation [1,3]. This case report describes a case of transomental hernia and characterizes its distinctive radiographic features.

## Case presentation

**Figure 1:**
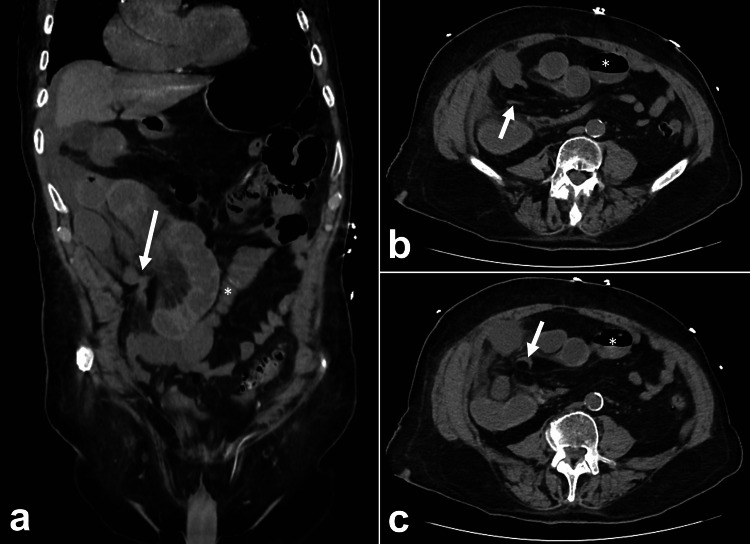
Computed tomography of the abdomen and pelvis with contrast showing multiple dilated, fluid-filled segments of the small bowel in the right hemiabdomen with surrounding ascites, intestinal edema, and mesenteric fat stranding. The cecum and ascending colon (asterisk) are displaced anteromedially (a) The coronal view shows the site of strangulation (arrow). The axial view shows the (b) proximal transition point (arrow) and (c) distal transition point (arrow) of the closed loop obstruction

An 82-year-old male presented with generalized abdominal pain that persisted over 24 hours. The patient was disoriented and a history could not be collected. Blood pressure was 160/75 mmHg, pulse rate 135/min, temperature 36.8°C, and SpO2 92%. On physical examination, the abdomen was distended and diffusely tender to palpation, with guarding in the right hemiabdomen but no rebound tenderness. Lactic acid was 4.6 mmol/L, and white blood cell count was 17.2 x 10^6^/mL. Computed tomography (CT) with contrast showed a closed loop obstruction along the right hemiabdomen as well as inflammatory features consistent with bowel ischemia (Figure 1).

Management

Exploratory laparotomy through a midline abdominal incision revealed bloody ascites and a 45 cm segment of gangrenous mid-ileum. There was a loop of bowel herniating through a defect in the greater omentum, with significant omental adhesions noted in the pericecal region. The gangrenous bowel segment was resected, and the healthy segments were anastomosed using gastrointestinal staples. The greater omentum was divided into two at the herniation site and adhesions were released to prevent recurrence of obstruction. The appendix was also noted to be absent, suggesting the patient had undergone a laparoscopic appendectomy in the distant past. After irrigation, the surgical site was closed, and the patient received transfusion of two units of packed red blood cells. The patient regained orientation to person and place on postoperative day three. However, he was unable to tolerate attempts at advancing oral intake. He experienced multiple episodes of aspiration pneumonitis, pulmonary edema, and respiratory decompensation and was therefore receiving nutrition via nasogastric tube feeding. He also experienced persistent and severe abdominal pain though were no signs of anastomosis failure or recurrence of bowel obstruction as the patient was passing flatus, having bowel movements, and tolerating tube feeding. Repeat abdominal imaging was unremarkable. On postoperative day 22, a joint decision was made by the patient and his family members to transition him to inpatient hospice care to focus on pain management. Two weeks after discharge to hospice, the patient died from respiratory failure.

## Discussion

SBOs are typically addressed conservatively with bowel rest, nasogastric tube decompression, and fluid resuscitation [1]. Although internal hernias are a rare etiology of SBO, they are associated with significant morbidity and mortality. The preoperative diagnosis of internal hernias is generally challenging due to patients presenting with nonspecific complaints such as abdominal pain or nausea and vomiting [1]. Vital signs and laboratory findings may also be within normal limits or borderline abnormal during the early stages of bowel ischemia [1,2]. Hence, an appreciation for the radiographic features of internal hernias may be helpful in deciding if surgical intervention is necessary.

Abdominal CT of patients with internal hernias classically shows SBO in an unusual location, with surrounding structures displaced from their expected anatomical positions [1-3]. In this case of transomental hernia, the abdominal CT showed ascites and edematous swelling compatible with bowel ischemia as well as a closed loop obstruction localized to the right paracolic gutter. Consequently, the cecum and ascending colon are displaced anteromedially. Delabrousse et al. reported a transomental hernia with a similar pattern of colonic displacement on CT [5]. Additional radiographic features characteristic of transomental hernia include engorgement and displacement of the mesenteric vascular pedicle as well as convergence and swirling of omental vessels around the hernia orifice [6,7].

While omental defects are commonly associated with major intraabdominal surgeries, this case demonstrates a potential association between laparoscopic appendectomy and internal hernia [1]. In addition, scarring and adhesions occur in about 5% of cases of laparoscopic appendectomies and significantly increase the risk of SBO [4]. The long-term risk of adhesion should be discussed with patients during preoperative evaluation for any intraabdominal surgeries [4,8]. While there are a few documented cases of transomental hernias occurring in patients with no prior surgical history, these are extremely rare [6,9]. Thus, reviewing a patient's surgical history, if available, may help with organizing the differential diagnoses.

In this case, the surgical team promptly proceeded with abdominal exploration given the clinical findings of peritonitis and radiographic evidence confirming an internal hernia. Although the immediate complications of bowel gangrene were prevented, the patient still experienced postoperative respiratory complications that eventually resulted in mortality. Further efforts are needed to improve the postoperative management of bowel gangrene resection, particularly in patients with a high risk of aspiration.

## Conclusions

In summary, it is crucial to recognize the radiographic signs of internal hernias and to conduct a thorough clinical examination in patients presenting with signs of bowel obstruction in order to expedite surgical intervention. A delayed diagnosis, especially in cases of transomental hernia, can significantly increase the risk of bowel ischemia, gangrene, and mortality.
